# Expression of TXLNA in brain gliomas and its clinical significance: a bioinformatics analysis

**DOI:** 10.1186/s41016-023-00341-4

**Published:** 2023-09-26

**Authors:** Bowen Hu, Desheng Chen, Yang Li, Shan Yu, Liangwen Kuang, Xinqi Ma, Qingsong Yang, Ke He, Yan Zhao, Guangzhi Wang, Mian Guo

**Affiliations:** 1 Department of Neurosurgery, The Second Affiliated Hospital of Harbin Medical University, 246 Xuefu Road, Nangang, Harbin, 150086 Heilongjiang Province China; 2 Department of Pathology, The Second Affiliated Hospital of Harbin Medical University, 246 Xuefu Road, Nangang, Harbin, 150086 Heilongjiang Province China

**Keywords:** Glioma, TXLNA, Gene expression, Bioinformatics analysis

## Abstract

**Background:**

To analyze the expression of TXLNA in brain gliomas and its clinical significance.

**Methods:**

Gene Expression Profiling Interactive Analysis（GEPIA）and Chinese Glioma Genome Atlas（CGGA）databases were retrieved as the methods. To assess the disparity between TXLNA expression in glioma and normal brain tissue. The Kaplan-Meier survival curve was employed to preliminarily evaluate the survival curves of the high and low expression groups, this was done for investigate the correlation between TXLNA expression level and the survival and prognosis of glioma. A Cox proportional regression risk model of multivariate nature was employed to evaluate the elements impacting the survival and prognosis of glioma. Gene pool enrichment analysis（GSEA）was used to investigate the related function of TXLNA in glioma. A Pearson correlation test and co-expression analysis were employed to identify the genes most associated with TXLNA expression.

**Result:**

The enrichment analysis results were observably enriched in signal pathways for instance the cell cycle and completion and coordination cascade pathways, and it is evident that high expression of TXLNA in gliomas is related to a poor survival and a bad patient prognosis, thus making it an independent prognostic factor for gliomas. Genes such as STK40 and R1MS1 are significantly correlated with TXLNA, playing a synergistic or antagonistic role.

**Conclusions:**

The prognosis of GBM patients is strongly linked to the high expression of TXLNA, which may be a viable therapeutic target for curbing cancer progression and creating new immunotherapies for GBM.

## Background

The two main subgroups of primary intracranial tumors, diffuse and non-diffuse gliomas, are the most prevalent [1]. Of all the diffuse gliomas diagnosed, glioblastoma (GBM) is the most deadly, accounting for 70–75% and having a median overall survival of only 14–17 months [2]. Currently, the treatment options for GBM are mainly surgical, with radiotherapy and chemotherapy added as an option. In addition, improvements in surgical microscopy, high-resolution imaging, fluorescence guided surgery, and neuronavigation are more and more used in the therapy of glioma [3]. However, due to its rapid spread, extensive infiltration and drug resistance, the postoperative recurrence rate is still high and the prognosis of patients is in a dismal state [4]. Thanks to cellular and biomolecular studies, the molecular processes of glioma and metastasis and novel targets for tumor therapy have been widely studied [5]. Currently, targeted therapy is a potential way to treat tumors. By exploring novel therapeutic objectives, targeted medications can be formulated and the outlook of patients enhanced. TXLNA (taxilin α) is a binding partner of the syntaxin family. Its alias in NCBI is also called interleukin 14 (IL-14), which is considered to be the key factor to coordinate the vesicular transport within cells. As we all know, vesicular transport is involved in multiple processes of eukaryotic cell proliferation, and its function also suggests that this gene may be involved in tumor expression [6]. More than that, a few Studies have established TXLNA’s existence and manifestation in hepatocellular carcinoma, renal cell carcinoma, and pancreatic tumor cells [7, 8]. In addition, Sueli M et al. conducted real-time fluorescence quantitative PCR analysis of TXLNA genes using different grade astrocytic tumor samples in an experiment, confirming that their expression was higher compared to non-tumor CNS tissues [9]. Until now, there has been a dearth of systematic and thorough research into the bioinformatics analysis of TXLNA about expression difference in brain gliomas, their clinical importance, and their mechanism of action, despite PCR analysis of TXLNA genes in glioma patients having been conducted. Therefore, the specific function of TXLNA in glioma, whether it accelerates the pathological process of glioma or regards as a target for clinical therapy, still requires a large amount of data analysis. This study explored the molecular and clinical characteristics of TXLNA in gliomas through bioinformatics analysis, and reached corresponding conclusions.

## Methods

### GEPIA

GEPIA, as a web server for cancer and normal gene expression and interaction analysis, is widely used by scholars around the world. It was used in this study to investigate the differential expression of TXLNA in glioma and normal brain tissue. Then we used one-way ANOVA to detect the survival curve of TXLNA high and low expression groups in glioma cells.

### CGGA

The Chinese Glioma Genome Atlas contains functional genomics data of 2000 Chinese glioma samples. This article primarily employs RNA sequencing data from genomic (1018 cases) and clinical information, both of which are found in the CGGA database. The clinical data include the gender, age, radiation and chemotherapy status of glioma patients, complete follow-up data, histopathological classification, different WHO grades, primary or recurrent gliomas. After the above data were downloaded, the sequencing data were batch corrected, and the clinical data such as expression data and survival data were further integrated. After filtering and screening, 749 patients were eventually included in our research.

### GSEA

GSEA Gene set Enrichment Analysis (GSEA) is a common and effective means to detect gene function and signaling pathways, and it is also a method to reveal genomic expression data. We divided the samples obtained from the CGGA database into two groups: high TXLNA expression and low TXLNA expression. P value less than 0.05, FDR value less than 0.25 is significant.

### Comprehensive analysis of transcriptome data of glioma samples from CGGA database

To discuss the prognostic value of TXLNA in GBM patients, the Kaplan Meier method and Cox regression model were utilized. GSEA was employed to forecast the related biological activities and pathways of TXLNA in glioma. Employing a Pearson correlation test and co expression analysis, the top five genes most closely relevant to TXLNA expression were identified, with the highest correlations being both positive and negative.

## Results

### Gliomas exhibiting a high expression of TXLNA are linked to a poor prognosis

**Fig. 1:**
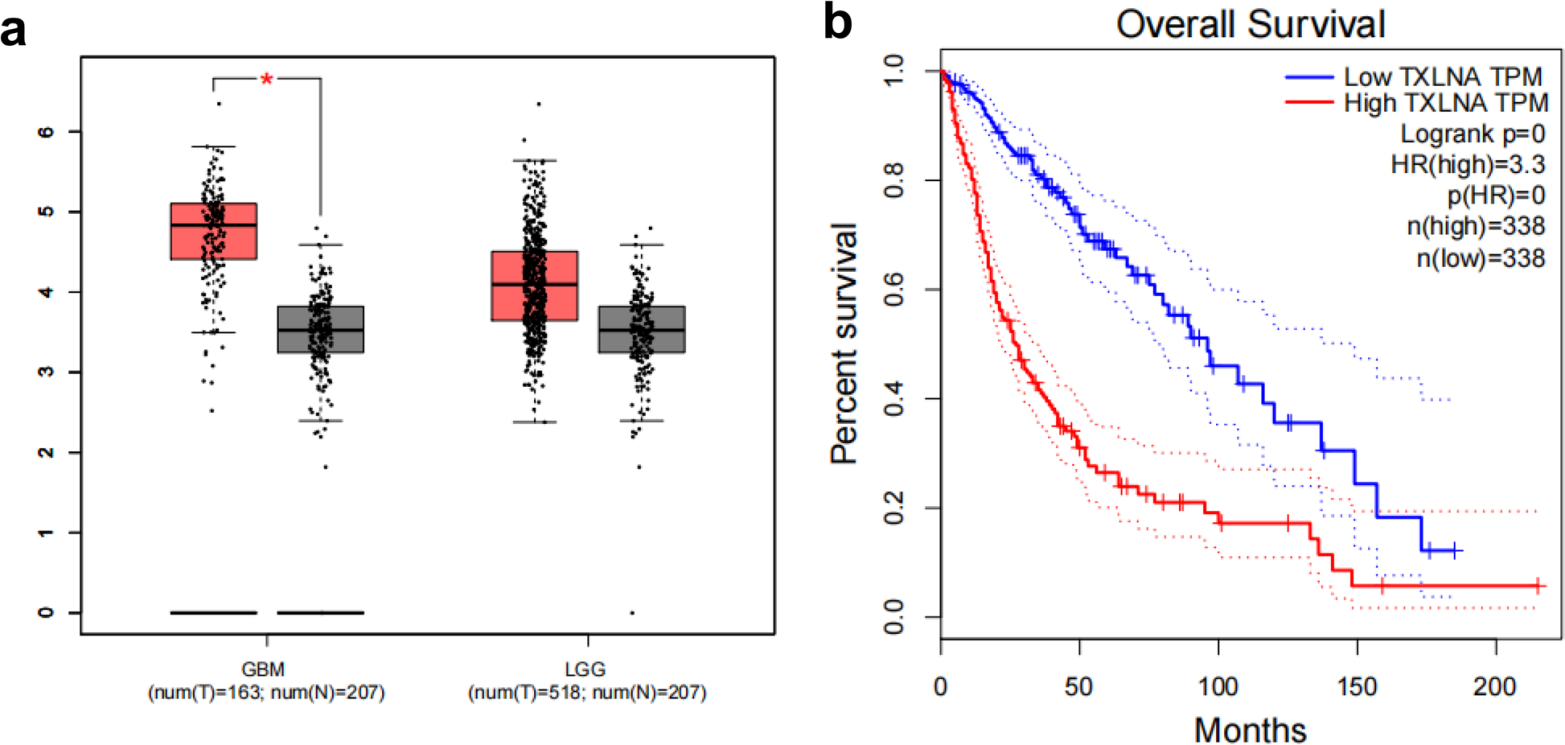
TXLNA is highly expressed in gliomas and is associated with poor prognosis. **A** The expression of TXLNA in GBM and LGG was higher than that in normal tissues in the GEPIA database. **B** Glioma patients with high TXLNA expression have shorter survival times than those with low expression in the GEPIA database

The expression of TXLNA in glioblastoma and low-grade glioma was observably higher than that of normal brain tissue (*P* < 0.05, Fig. 1a). Moreover, as malignancy deepens, the expression of TXLNA is also rising. A poor prognosis in glioma patients is associated with a high TXLNA expression (*p* < 0.05, Fig. 1b).

### TXLNA is highly expressed in gliomas and reduces overall survival time

**Fig. 2:**
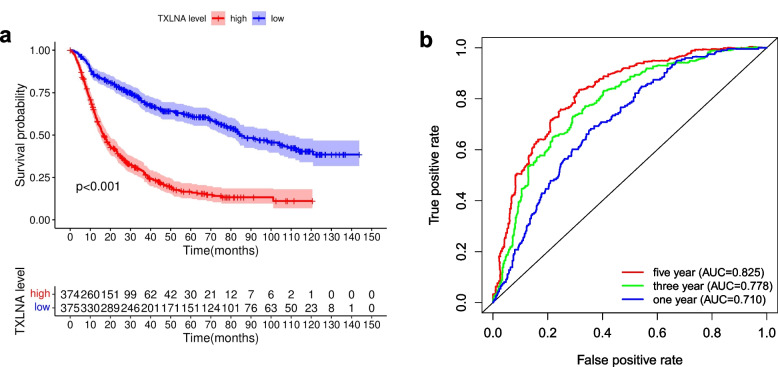
The Kaplan-Meier survival curve and The ROC curve. **A** Highly expressed TXLNA leads to shorter overall survival time for patients with glioma. **B** ROC curve shows that using TXLNA as a prognostic marker is valuable

According to the expression difference of TXLNA, We split the samples into two distinct groups, one with high TXLNA expression and the other with low TXLNA expression, to explore the effects of TXLNA on glioma patients. Survival kits and questionnaires were employed to assess the distinctions between the two groups, and the outcomes showed that the survival rate of those in the high and low expression groups had decreased over time, and the survival disparity between the two groups was highly significant (*p* < 0.001). The TXLNA high expression group had a 5-year survival rate of 16.0%(95% CI [0.1219–0.211]), while the TXLNA low expression group had a 5-year survival rate of 61.4%(95% CI [0.563–0.670]) (Fig. 2a), as indicated by statistical analysis. Verification of TXLNA expression levels' reliability in predicting patient survival time was further accomplished by employing the receiver operating characteristic curve (ROC). The prognosis model's 1-year, 3-year, and 5-year ROC curves all had AUCs that surpassed 0.7 (Fig. 2b). Moreover, the area beneath the ROC curve (AUC) was also higher than 0.7, suggesting TXLNA expression is reliable in forecasting survival time.

### The prognosis of glioma patients can be predicted independently by the expression of TXLNA

**Fig. 3:**
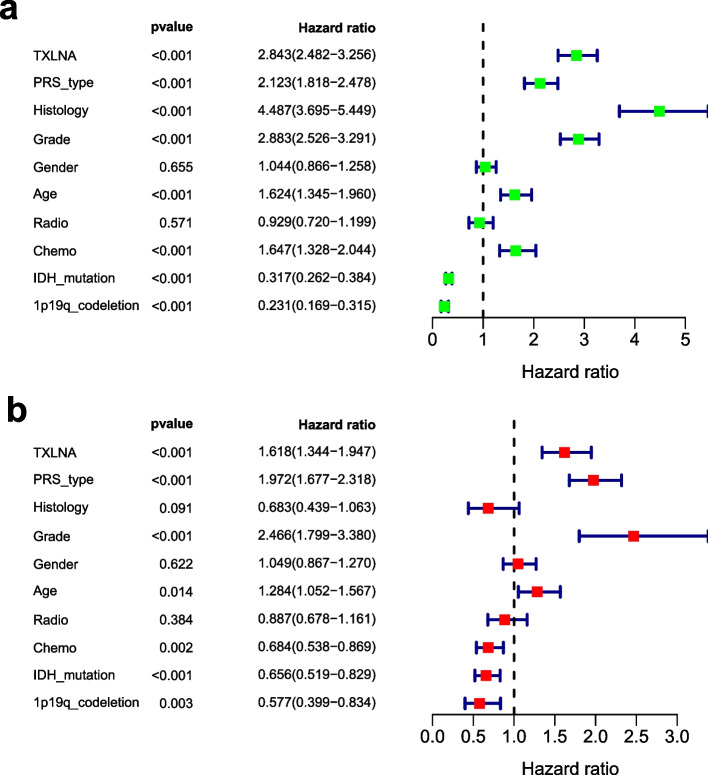
Relationship between clinical features and prognosis of patients with glioma. **A** Univariate analysis. **B** Multivariate analysis

We compared TXLNA gene expression, age, and IDH mutations to survival time and survival status in order to assess if TXLNA could be regarded as a risk factor for predicting GBM patients' prognosis. A Cox regression model was used to evaluate the relationship between TXLNA expression, clinical characteristics and prognosis, from which we found that tumor recurrence, age, and IDH mutation were closely related to the survival rate of GBM patients. Furthermore, we confirmed that TXLNA could be regarded as an independent risk factor in GBM patients by univariate and multivariate Cox analysis (Fig. 3).

### Relationship between TXLNA and different clinical characteristics

**Fig. 4:**
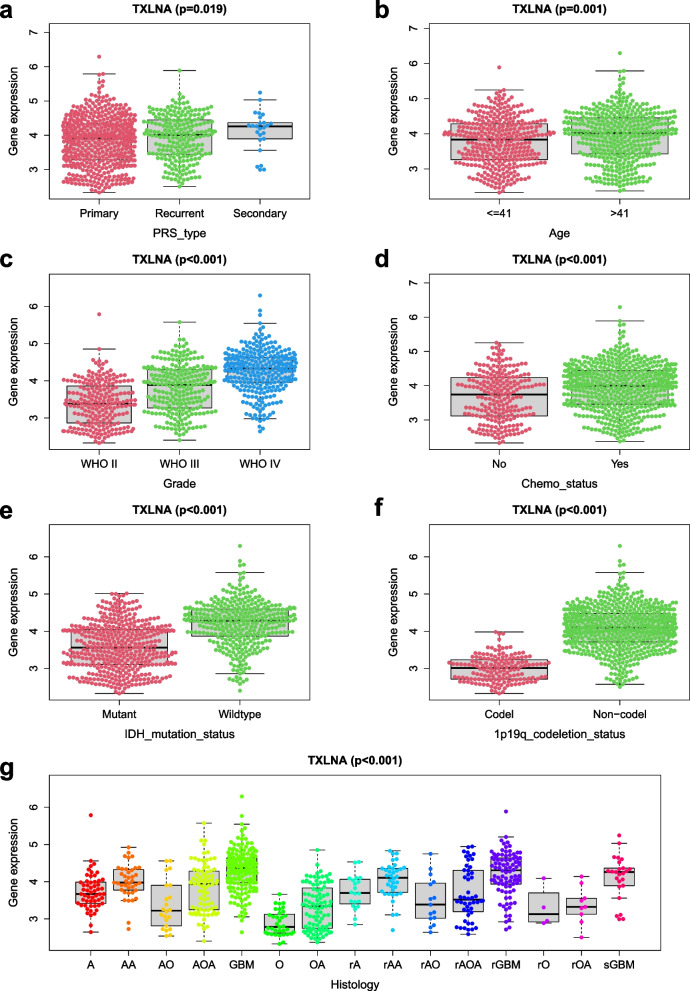
Relationship between TXLNA and clinical characteristics. **A** PRS type. **B** Age. **C** Grade. **D** Chemotherapy. **E** IDH mutation. **F** 1p/19q codeletion. **G** Histology

Through the analysis of previous studies, we concluded that TXLNA is an independent prognostic factor for GBM patients. The clinical features of it are also closely connected with the prognosis. To further explore the relationship between TXLNA and clinical factors, the Wilcox test and Kruskal method were used by us. A correlation between TXLNA expression and tumor grade was demonstrated, with the expression of TXLNA gradually augmenting with the augmentation of tumor grade (*p* < 0.001). Additionally, the expression of TXLNA was linked to the age of the patients, with those aged > 41 years exhibiting a greater gene expression than those aged < 41 years (*p* = 0.001). Then, the correlation between TXLNA expression and the IDH mutation means that there is a link. What is more, TXLNA expression in wild-type tumors of IDH was distinctly higher than that of mutant genes (*p* < 0.001). TXLNA expression and recurrent status were also proved closely related by analysis (*p* = 0.019), with primary tumors displaying a lesser TXLNA expression than recurrent status. TXLNA expression was correlated with 1p19q deletion, and its expression in 1p19q deletion was lower than that in wild type without 1p19q deletion (*p* < 0.001); Finally, TXLNA expression is also associated with chemotherapy, and analysis suggests that TXLNA expression in non-chemotherapy recipients is lower than that in chemotherapy recipients (*p* < 0.001)(Fig. 4a–g).

### The potential pathway of TXLNA in regulating the malignant biological behavior of glioma

**Fig. 5:**
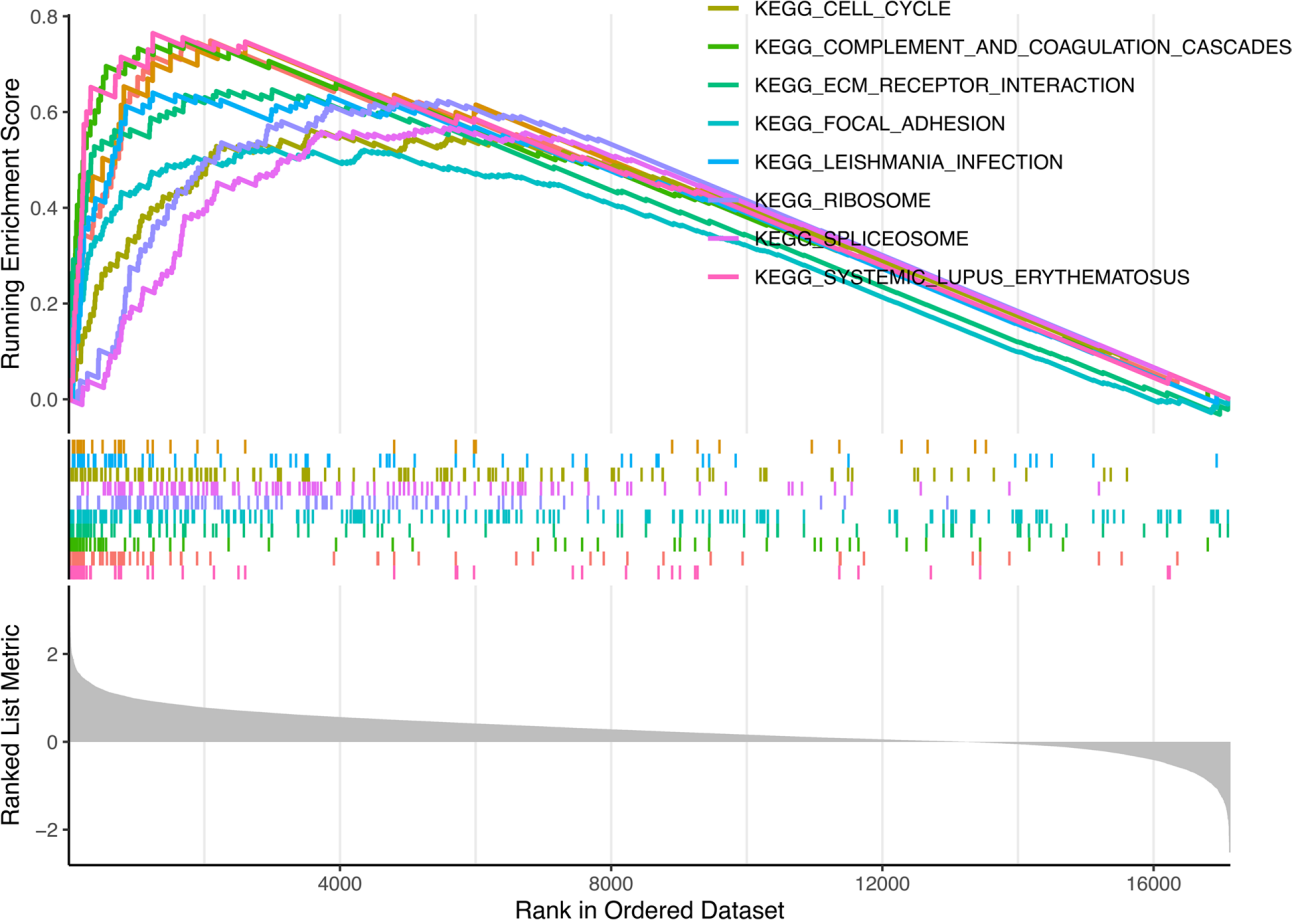
Significantly enriched pathways with multiple GSEA

Employing the GSEA technique, we pinpointed the biological functions linked to TXLNA. By identifying TXLNA enrichment pathways, we can indirectly understand what pathways TXLNA regulates to affect the occurrence of glioma. We divided the sample data into two groups according to the high and low expression of TXLNA. The consequences showed that the expression of TXLNA was relevant to a variety of tumor interrelated pathways, including the cell cycle path, completion and coaggregation casca path, receiver interaction path, focal adhesion path, leishmania infection path, ribosome path, splice some path, and lupus erythematosus path (Fig. 5). Therefore, these pathways can be considered as potential pathways for TXLNA regulation in glioma.

### Coexpression analysis

**Fig. 6:**
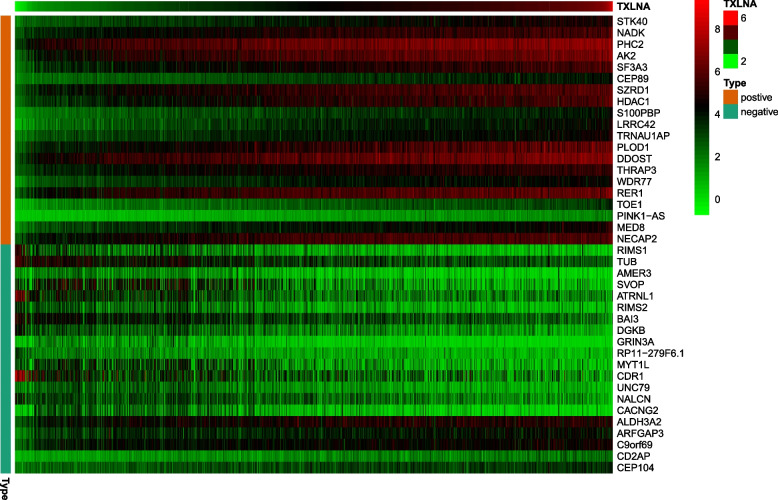
Gene expression heat map and correlations for TXLNA co-expressed genes

**Fig. 7:**
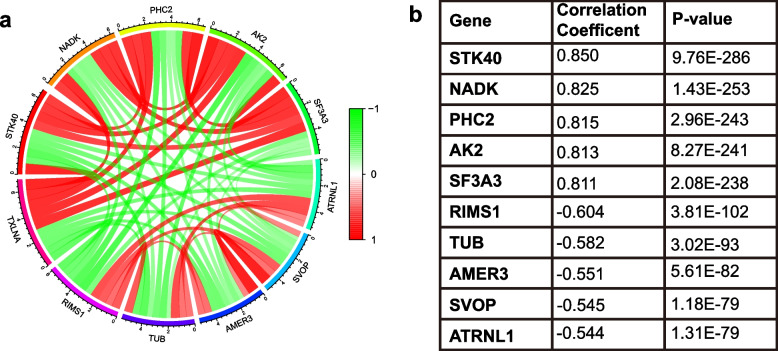
The co-expressed network of TXLNA. **A** The circos plot was constructed by 10 genes most related to TXLNA expression, including 5 positively related genes and 5 negatively related genes (*p* < 0.05). **B** Co-expression relationship of 10 genes in TXLNA

Pearson’s correlation test was employed to further elucidate TXLNA’s part in the pathogenesis of gliomas, and TXLNA related genes were pinpointed. Filter and screen out the 10 genes most relevant to TXLNA, including the first 5 genes that are positively correlated, namely STK40, NADK, PHC2, AK2, and SF3A3, as well as the first 5 genes that are negatively correlated, including R1MS1, TUB, AMER3, SVOP, and AJRNL1. From Figs. 6 and 7, it can be seen that TXLNA perhaps accelerate the expression of positively correlated genes, or these co expressed positive correlated genes together with TXLNA affect specific pathophysiological processes, thereby promoting the occurrence and progress of GBM. Contrarily, TXLNA may inhibit the expression of negatively correlated genes, or their functions may be antagonistic to each other.

## Discussion

TXLNA is a significant cytokine, It is also known as interleukin 14(IL-14) in NCBI, meanwhile, it is also went by the name of high-molecular-weight B cell growth factor(HMW-BCGF). It is primarily generated by T lymphocytes and can trigger the proliferation of activated B cells, impede immunoglobulin secretion, and selectively enlarge certain B cell subsets. Studies of antibodies to HMW-BCGF and its receptors have revealed that, despite its production by T cells and some malignant B cells, it primarily impacts both normal and malignant B cells [10]. Confirmed to be involved in Ca^2+^ dependent exocytosis of neuroendocrine cells, TXLNA has a vital role in intracellular material transport, which is essential for tissue structure and cell function. The soluble N-ethylmaleimide-sensitive factor attachment protein receptor (SNARE) mechanism plays a dominant role in intracellular vesicle transport. Although its specific mode of action and function are still unknown, TXLNA, as a novel binding partner of the synthetic protein family, has been approved to be involved in the control of the formation of SNARE complexes [6]. This function of TXLNA suggests that it may participate in the gene expression of tumor cells. Its role also could be to either accelerate or impede the growth and emergence of tumor cells.

However, reports have shown that TXLNA has different functions in different types of tumors. TXLNA, for example, is a cancer suppressor gene in pancreatic cancer, yet it is a promoter of tumors in hepatocellular carcinoma and renal cell carcinoma, thus inducing their emergence and development. It was proposed by Natsuko Ohtomo et al. that the TXLNA expression is linked to the augmented proliferation and poorly differentiated histological evaluation of hepatocellular carcinoma [7]. Tomoko Mashidori 1 et al. found that the metastatic and invasive capacity of renal cell carcinoma was revealed to be augmented by a high expression of TXLNA [11]. However, not high TXLNA expression in all tumors is associated with poor prognosis. In the research on pancreatic cancer, it was concluded that the high expression of TXLNA suggests a favorable prognosis for patients [8]. In addition, there are relevant studies in non-solid tumors. Studies have established that in invasive B cell non-Hodgkin’s lymphoma (NHL-B), both autocrine and paracrine IL-14 may be an important factor in its rapid proliferation [12].

Nevertheless, the role of TXLNA in glioma is still a mystery. To uncover its expression and clinical importance, we conducted bioinformatics studies on GEPIA and CGGA databases to compare the expression and prognostic analysis of TXLNA. A marked divergence in TXLNA expression between glioma and normal brain tissue was uncovered by our findings, and a negative relationship between TXLNA expression and OS in glioma patients was also observed. The ROC curve confirms that TXLNA expression is reliable in predicting survival time. The occurrence and development of tumors, though not due to a single cause, is still influenced by many factors; thus, it is uncertain if TXLNA can be a distinct risk factor for glioma patients. Furthermore, the development of glioma is not caused by a single gene, but rather by the disruption of the equilibrium between multiple carcinogenic and tumor suppressor elements. Unveiling the gene’s mechanism of action in glioma necessitates the examination of other genes associated with it, as well as the cellular pathways that are implicated.

Therefore, we used Cox regression model to conduct univariate and multivariate analysis, and found that TXLNA can still be an independent risk factor to forecast the prognosis of GBM patients after eliminating the impact of other elements. In addition, other clinical features, including PRS type, grade, age, chemotherapy, IDH mutation status, and 1p19q co deletion status, could also be independent risk factors. Utilizing gene set enrichment analysis (GSEA), we investigated TXLNA’s expression and discovered its association with multiple tumor-related pathways, such as the cell cycle path, completion and coaggregation cascade path, receiver interaction path, focal adhesion path, leishmania infection path, ribosome path, splice some path, and lupus erythematosus path. Recent articles have explored the correlation between certain pathways and glioma’s biological behavior. COL1A2 inhibition attenuates GBM proliferation which is depended on accelerating cell cycle arrest [13]. The casca pathway of coagulation could be a critical factor in immunotherapy and anti-cancer immunity, not only aiding in the elimination of tumors but also stimulating tumor expansion [14]. The Kangliu pill(KLP) has anti-tumor activity in glioma, which may be related to receptor interaction pathway [15]. The adhesion signaling pathway is activated by mda-9/syntenin, which in turn induces glioma migration through adhesion kinase (FAK) [16]. Pathways of this kind are a major factor in the pathology of glioma, stimulating malignant biological activities such as proliferation, migration, and invasion of glioma cells. However, there is still a lack of laboratory data to support the relationship between some pathways and TXLNA expression in glioma cells or tumor cells.

Finally, Pearson correlation test and co expression analysis were employed to identify the 10 genes most relevant with TXLNA expression, of which 5 were found to be positively correlated—STK40, NADK, PHC2, AK2, and SF3A3. These genes' pathophysiological processes have been established to be strongly correlated with the biological activity of tumors. Belonging to the Tribbles pseudokinases family, STK40 is seen as a marker of cancer progression [17]. NADK and KHK, two genes responsible for metabolic enzymes, are inactivated in animal models, thus inhibiting the growth of tumors with KRAS mutations [18]. In an epigenetic study of prostate cancer cells, it was found that R1881 treated cells had higher PHC2 methylation [19]. This indicates that PHC2 methylation is associated with the occurrence and progress of prostate cancer. The over-expressed of AK2 and other genes was reported to be a factor in the growth of breast cancer that was invasive [20]. The interplay between CircSCAP and SF3A3 inhibits the malignancy of non-small cell lung cancer by activating p53 signaling pathways [21]. In addition, we have also screened five negatively correlated genes, including RIMS1, TUB, AMER3, SVOP, and ATRNL1. Among them, the expression of RIMS1 was also distinctly downregulated in Adamantinomatous craniopharyngioma cancer samples [22]. Moreover, TUB low expression in circulating tumor cells inversely influenced patients’ OS as independent prognostic factors [23]. What is more, AMER3 variants modify the U-shaped association of urinary total hydroxyphenanthrene with fasting plasma glucose [24], however, the association of this gene and tumor is rarely reported. A study conducted by Jingwei Zhao identified five Differential methylated genes as critical prognostic biomarkers in GBM progression, including the SVOP [25]. A retrospective study of 454 cases with solid pseudopapillary neoplasm of the pancreas uncovered that ATRNL1 is one of the top three frequently mutated genes [26]. In general, the occurrence and progress of GBM is a very intricate biological course. TXLNA’s involvement in the glioma regulatory network is demonstrated through its interaction with cancer genes and cancer suppressor genes.

## Conclusion

TXLNA expression in glioma tissues is far greater than in normal tissues, and its upsurge is associated with a reduced likelihood of survival and a dismal outlook. It can be employed as a distinct prognostic indicator for glioma patients. Its mechanism of action is significantly enriched in signal pathways such as the cell cycle path and the completion and coordination cascade path. STK40, NADK, PHC2, AK2, and SF3A3 are positive correlation genes for TXLNA, while R1MS1, TUB, AMER3, SVOP, and AJRNL1 are negative correlation genes for TXLNA. Our findings reveal a correlation between GBM and malignant clinical features. Our discoveries furnish novel diagnostic and prognostic indicators for further investigation.

## Data Availability

All the data in this study were obtained from Chinese Glioma Genome Atlas dataset (CGGA; http://www.cgga.org.cn/).

## Abbreviations

GEPIA: Gene Expression Profiling Interactive Analysis
CGGA: Chinese Glioma Genome Atlas
GSEA: Gene pool enrichment analysis
GBM: Glioblastoma
